# A study of viral pathogens in bat species in the Iberian Peninsula: identification of new coronavirus genetic variants

**DOI:** 10.1080/23144599.2022.2139985

**Published:** 2022-11-03

**Authors:** Alberto Moraga-Fernández, Marta Sánchez-Sánchez, João Queirós, Ana M. Lopes, Joaquín Vicente, Xosé Pardavila, Jorge Sereno-Cadierno, Paulo C. Alves, José de la Fuente, Isabel G. Fernández de Mera

**Affiliations:** a Institute for Game and Wildlife Research, IREC (CSIC-UCLM-JCCM), SaBio Research Group, Ciudad Real, Spain; b CIBIO, Centro de Investigação em Biodiversidade e Recursos Genéticos, InBIO Laboratório Associado, Campus de Vairão, Universidade do Porto, Vairão, Portugal; c BIOPOLIS Program in Genomics, Biodiversity and Land Planning, CIBIO, Campus de Vairão, Vairão, Portugal; d Estação Biológica de Mértola (EBM), CIBIO, Praça Luís de Camões, Mértola, Portugal; e Sorex, Ecoloxía e Medio Ambiente S.L., Santiago de Compostela. A Coruña, Spain; f Departamento de Biologia, Faculdade de Ciências, Universidade do Porto, Porto, Portugal; g Center for Veterinary Health Sciences, Oklahoma State University, Stillwater, OK, USA

**Keywords:** Bats, coronavirus, emerging pathogens, SARS-CoV-2, zoonosis, wildlife

## Abstract

Bats have long been associated with multiple pathogens, including viruses affecting humans such as henipaviruses, filoviruses, bunyaviruses and coronaviruses. The alpha and beta coronaviruses genera can infect most mammalian species. Among them, betacoronavirus SARS-CoV, MERS-CoV and SARS-CoV-2, which have caused the three major pandemics in the last two decades, have been proposed to originate in bats. In this study, 194 oral swabs from 22 bats species sampled in 19 locations of the Iberian Peninsula were analysed and characterized by three different PCR tests (coronavirus generic real-time RT-PCR, multiplex conventional PCR, and SARS-CoV-2 specific real-time RT-PCR) to detect bat coronaviruses. Screening with coronavirus generic PCR showed 102 positives out of 194 oral swabs analysed. Then, metabarcoding with multiplex PCR amplified 15 positive samples. Most of the coronaviruses detected in this study belong to alphacoronavirus (α-CoV) genus, with multiple alphacoronaviruses identified by up to five different genetic variants coexisting in the same bat. One of the positive samples identified in a *Miniopterus schreibersii* bat positive for the generic coronavirus PCR and the specific SARS-CoV-2 PCR was classified as betacoronavirus (-CoV) through phylogenetic analysis. These results support the rapid evolution of coronaviruses to generate new genomic potentially pathogenic variants likely through co-infection and recombination.

## 1. Introduction

Chiroptera order is one of the most abundant, diverse, and widespread group of mammals, comprising approximately 1,200 species and representing almost 25% of the class Mammalia. Bats are the second biggest order of mammals and the only ones capable of flying. Thirty-one species of this order are native to the Iberian Peninsula. In recent years, bats have been of particular interest not only for their ecological implications, but also for public health reasons, as they are considered to play an important role in the emergence and transmission of zoonotic pathogens [1]. These animals are vectors and natural reservoirs for a wide range of microorganisms with the ability to cross the species barrier, which makes them potential sources of zoonotic pathogens.

Bats have been associated with highly pathogenic human viruses, acting as hosts for recombination, transmission and spread of these pathogens. Examples include lyssaviruses (rabies virus), henipaviruses (Nipah virus and Hendra virus), filoviruses (Marburg virus, Ebola virus and Mengla virus), bunyaviruses (Ahun virus) [1–3] and coronaviruses, putative precursors of the pandemic-associated viruses SARS-CoV [4–6], MERS-CoV [7–10] and more recently SARS-CoV-2 [11–14]. SARS-CoV emerged in 2002–2003 in the Guangdong province, China, and bats were identified as the reservoir and probable source of this emerging disease-causing pathogen [4,5]. Ten years later, in 2012, MERS-CoV emerged in the Middle East and although in this case camels were identified as the source for human infection, closely related viruses to MERS-CoV were also identified in bats [15]. The last COVID-19 pandemic caused by SARS-CoV-2 probably had its origin in wild animals, and an association between bats and this emerging zoonotic disease has been established [11–14]. For this reason, the surveillance of bats and other wild animals that could act as potential reservoirs for these pathogens has been promoted.

Alphacoronaviruses (α-CoV) and Betacoronaviruses (β-CoV) infect several mammalian species, including humans, bats and pigs, while Gammacoronaviruses (-CoV) and Deltacoronaviruses (δ-CoV) infect birds, wild felines, pigs and some marine mammalian species [16,17]. Both α-CoVs and β-CoVs have been identified in bats from different European countries such as Italy [18–24], France [25,26], United Kingdom [27,28], Germany [29–31], Romania [29], the Netherlands [29,32], Ukraine [29], Finland [33], Denmark [34,35], Hungary [36], Bulgaria [37], Slovenia [6], Luxembourg [38] and Switzerland [39]. In some cases, both α-CoVs and -CoVs genera coexist in the same animal, as it has been detected in the Iberian Peninsula or other locations [40,41].

One of the priorities of the World Health Organization (WHO) is the surveillance of emerging diseases and the development of preventive and control interventions. The WHO gives priority to emerging diseases associated with public health risk due to their epidemic potential and/or limited or insufficient control interventions. Some of the viruses associated with bats (https://www.who.int/activities/prioritizing-diseases-for-research-and-development-in-emergency-contexts) are associated with these prioritized emerging diseases.

The aim of this study was the detection and classification of coronaviruses present in Iberian bat species to advance knowledge on their prevalence, and to improve control and surveillance measures for potentially new emerging zoonotic coronaviruses.

## 2. Materials and methods

### 2.1. Ethical statement

The authors confirm that the ethical policies of the journal have been adhered to. Samples were obtained and processed following standard operating procedures with the appropriate approval of the Ethical and Scientific Committees (AUES/CYL/208bis/2021; AUF/202170016; EB/039/2019-1).

### 2.2. Sample collection

**Figure 1.**
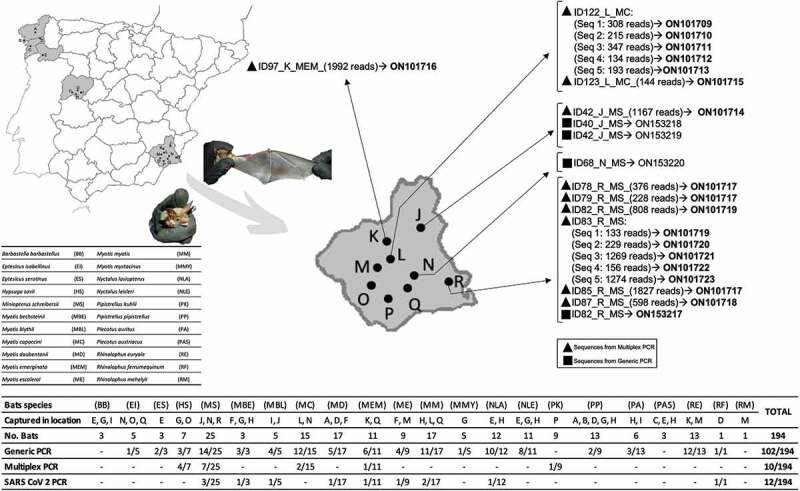
Distribution of captured bat species in the sampled areas showing the results of coronavirus detection.

To detect the presence and distribution of the different coronaviruses in Spanish bats, 194 animals belonging to 22 species were captured with mist nets and harp traps setting up in activity areas (ponds, riverine habitats, etc.) and close to roost [42], at 19 sites distributed by five provinces of Spain during the summer of 2020 (Table 1; Figure 1). Capture, handling, and sampling of animals were carried out by trained personnel, complying with all relevant national guidelines and institutional policies, and in strict accordance with good animal practices. Moreover, instruments were sterilized between captures, and personnel was protected with gloves and facemasks, following the specific regulations of working with bats in a pandemic scenario, according to the Spanish Association for the Conservation and Study of Bats (www.secemu.org). Each animal was maintained in individual sterilized cotton bags to avoid sample contamination before morphologically identified by expert personnel using taxonomic keys [43], and straightaway oro-pharyngeal swabs were collected; finally, the animals were released.

**Table 1.** Sample collection distribution and classification.

Latitude	Longitude	Province	Location (Location Ref.)		Bat species (Abbreviation and sample number)		
42.94588	−8.53053	La Coruña	Chaian	(A)	*Pipistrellus pipistrellus*	(PP)	3
					*Myotis daubentonii*	(MD)	10
42.59875	−8.959847	La Coruña	Mirandela	(B)	*P. pipistrellus*		3
42.35164	−8.517887	Pontevedra	Chaín	(C)	*Plecotus austriacus*	(PAS)	1
42.77171	−8.09657	Pontevedra	Arnego	(D)	*M. daubentonii*		6
					*P. pipistrellus*		3
					*Rhinolophus ferrumequinum*	(RF)	1
41.99619	−8.02963	Ourense	Meás	(E)	*Barbastella barbastellus*	(BB)	1
					*Eptesicus serotinus*	(ES)	3
					*Nyctalus lasiopterus*	(NLA)	2
					*Nyctalus leisleri*	(NLE)	1
					*P. austriacus*		1
40.56172	−6.14181	Salamanca	El Cabaco	(F)	*Myotis bechsteinii*	(MBE)	1
					*M. daubentonii*		1
					*Myotis escalerai*	(ME)	1
40.59649	−6.02628	Salamanca	Las Fuentes	(G)	*B. barbastellus*		1
					*Hypsugo savii*	(HS)	3
					*M. bechsteinii*		1
					*Myotis mystacinus*	(MMY)	5
					*N. leisleri*		2
					*P. pipistrellus*		3
40.54936	−6.0606	Salamanca	Fuente Castaño	(H)	*M. bechsteinii*		1
					*Myotis myotis*	(MM)	2
					*N. lasiopterus*		10
					*N. leisleri*		8
					*P. pipistrellus*		1
					*Plecotus auritus*	(PA)	1
					*P. austriacus*		1
40.57282	−5.95134	Salamanca	Fuente del Cerezo	(I)	*B. barbastellus*		1
					*Myotis blythii*	(MBL)	1
					*P. auritus*		5
38.20813	−1.11898	Murcia	La Almagra	(J)	*Miniopterus schreibersii*	(MS)	5
					*M. blythii*		4
38.14656	−1.37617	Murcia	Ricote	(K)	*Myotis emarginata*	(MEM)	10
					*Rhinolophus euryale*	(RE)	8
37.99471	−1.48971	Murcia	Sima del Almez, Pliego	(L)	*Myotis capaccini*	(MC)	10
					*M. myotis*		12
37.97833	−1.50172	Murcia	Sima de la Higuera	(M)	*M. escalerai*		8
					*R. euryale*		5
					*Rhinolophus mehelyii*	(RM)	1
37.81409	−1.26656	Murcia	Minado de Carrascoy	(N)	*Eptesicus isabellinus*	(EI)	3
					*M. schreibersii*		8
					*M. capaccini*		5
37.88693	−1.59861	Murcia	Malvariche	(O)	*E. isabellinus*		1
					*H. savii*		4
37.85594	−1.51191	Murcia	Huerta Espuña	(P)	*Pipistrellus kuhlii*	(PK)	9
37.80680	−1.27157	Murcia	Escobar	(Q)	*E. isabellinus*		1
					*M. emarginata*		1
					*M. myotis*		3
37.79657	−0.90413	Murcia	Torre Pacheco	(R)	*M. schreibersii*		12

### 2.3. RNA extraction

The oropharyngeal swabs were preserved in Tri-Reagent (Sigma-Aldrich, Burlington, MA, USA) at −80°C. RNA extraction was conducted in a biosafety level 3 cabinet. Tri-Reagent was used for nucleic acid extraction according to the manufacturer’s instructions.

### 2.4. Gene amplification and sequence analyses

Samples were analysed based on the three techniques detailed below.

#### 2.4.1. Generic real-time RT-PCR

A described real-time RT-PCR [44] was user for the generic screening of coronaviruses. The 194 RNA samples were analysed by a real-time RT-PCR for the generic detection of coronaviruses, targeting the most conserved fragment belonging to ORF1b region and amplifying a fragment of 179 bp [44]. Sequences of the primers are described in Table 2. All reactions were carried out on a CFX96 Touch Real Time PCR Detection System (Bio-Rad, Hercules, CA, USA) using the iTaq Universal One-Step RT-qPCR kit with SYBR Green (Bio-Rad, Hercules, CA, USA). Volume reaction was adjusted to 15 µl and included 7.5 µl reaction mix, 1 µl of each primer at 10 µM, 0.25 µl of iScript reverse transcriptase, 2 µl of RNA and 3.25 µl of nuclease free water to complete the reaction volume. Conditions of the real-time RT-PCR were as follows: reverse transcription was carried out at 50°C for 15 min, followed by polymerase activation and first denaturation at 95°C for 1 min and by 40 cycles of 95°C for 10 sec, 50°C for 20 sec and 60°C for 20 sec. Melting curve analysis was performed by heating the samples to 95°C at 0.5°C increments every 6 sec.

**Table 2.** Primers used for partial gene amplifications.

PCR	Primer	Sequence 5’- 3’	Reference
Coronavirus generic	11_FW	TGATGATGSNGTTGTNTGYTAYAA	[55]
	13_RV	GCATWGTRTGYTGNGARCARAATTC	
Multiplex	Cor_F_1	TATTTKAARCCWGGYGGTAC	This study
	Cor_F_2	TATGTNAARCCHGGYGGTAC	
	Cor_F_3	TACGTNAAACCTGGWGGTAC	
	Cor_F_4	TACTTWAAACCWGGTGGTAC	
	Cor_F_5	TATATRAARCCTGGTGGTAC	
	Cor_F_6	TATGTTAARCCWGGTGGAAC	
	Cor_F_7	TATGTNAARCCWGGHGGCAC	
	Cor_R_1	GAACARAAYTCATGNGGTCC	
	Cor_R_2	GAGCARAAYTCRTGAGGTCC	
	Cor_R_3	GAACAAAAYTCATGWGGACC	
	Cor_R_4	GAACARAAYTCATGTGGCCC	
	Cor_R_5	GAACARAAYTCATGDGGGCC	
SARS-CoV 2	N1_F	GACCCCAAAATCAGCGAAAT	https://www.cdc.gov/coronavirus/2019-ncov/lab/virus-requests.html
	N1_R	TCTGGTTACTGCCAGTTGAATCTG	
	N1_P	FAM-ACCCCGCATTACGTTTGGTGGACC-BHQ1	
	N2_F	TTACAAACATTGGCCGCAAA	
	N2_R	GCGCGACATTCCGAAGAA	
	N2_P	FAM-ACAATTTGCCCCCAGCGCTTCAG-BHQ1	

**Figure 2.**
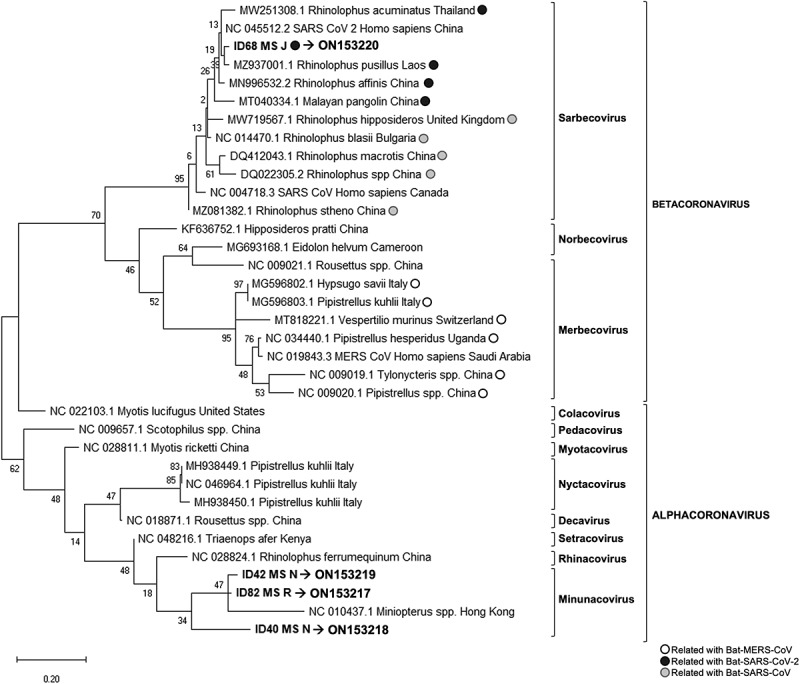
Phylogenetic tree generated for the 118 bp fragment from the coronavirus generic PCR (ORF1b region).

PCR products were sequenced and aligned with reference sequences obtained from GenBank using ClustalW. Reference sequences included different bat coronavirus, as well as SARS-CoV, SARS CoV-2 and MERS-CoV strains isolated from humans. The fragment analysed was 118 bp, and a phylogenetic tree was generated using MEGA version 10 (http://www.megasoftware.net) with the maximum likelihood method with Tamura 3-parameter model [45]. Model of the sequence evolution was selected based on Corrected Akaike Information Criterion (cAIC) and Bayesian Information Criterion (BIC); the model with lowest values of cAIC and BIC was chosen. Bootstrap confidence limits were calculated based on 1000 replicates; branch numbers in the tree indicate bootstrap results (Figure 2).

#### 2.4.2. Conventional multiplex PCR

**Figure 3.**
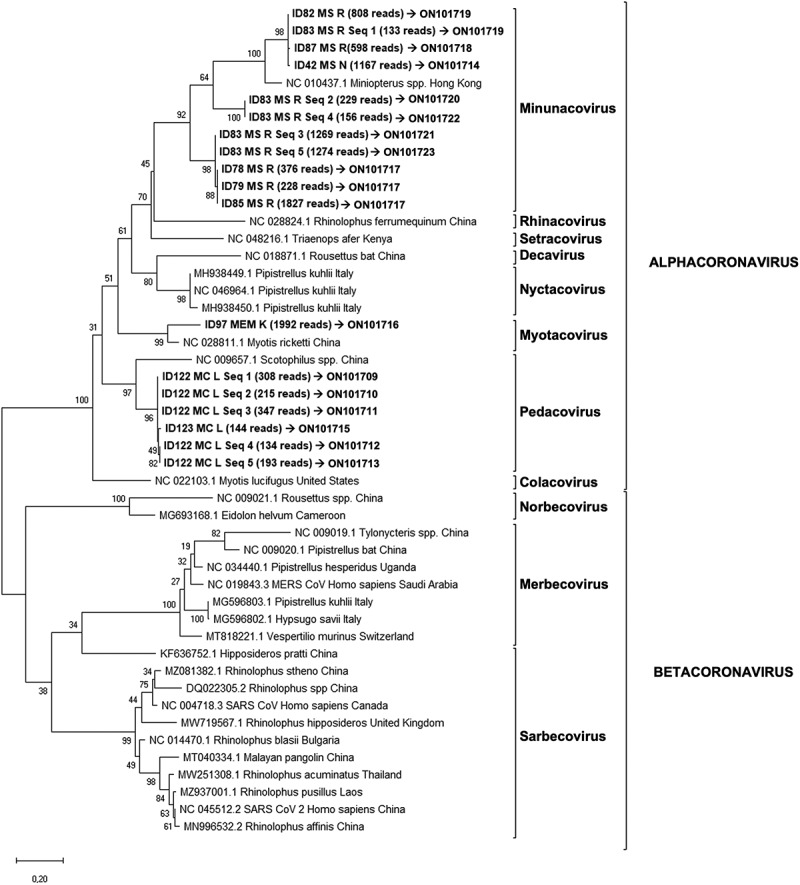
Phylogenetic tree generated for the 381 bp fragment from the coronavirus Multiplex-PCR (ORF1ab region).

In addition, a novel conventional multiplex PCR designed for this study was performed to characterize and sequence the community of coronaviruses co-infecting a bat, by using a multiplex primer approach and a high-throughput sequencing technology. To design general primers for coronaviruses, 65 genomes available in GenBank for different animal species (list of genomes in Supporting Information) were downloaded and aligned with Clustal X, using the program UGENE (http://ugene.net). Primer sites were selected in two of the most conserved regions of the genomes, amplifying a fragment of 422 bp within the ORF1ab polyprotein gene, between the nucleotide positions 14798 and 15219 of the reference genome sequence NC_048212.1. This region showed a high divergence among genomes in preliminary phylogenetic analysis. A set of 12 primers (7 forward and 5 reverse) were designed to potentially amplify the 65 genomes, following the default conditions of the program UGENE, and ensuring that no more than five degenerated bases were added at each primer to minimize cross amplifications with bat genome (Table 2). Reverse transcription was performed with the First-Strand cDNA Synthesis Kit (NZYTech, Lisbon, Portugal) according to the manufacturer’s instructions. PCR reactions were carried out in a T100 Thermal cycler (Bio-Rad, Hercules, CA, USA) using the Multiplex PCR Kit (QIAGEN, Hilden, Germany) and the mix of primers (0.4 µl of each primer at 100 µM in a final volume of 50 µl). Avian and human positive controls (identified as APC and HPC, respectively) were used to check and optimize the PCR conditions. Volume reaction was adjusted at 10 µl, and included 5 µl reaction mix, 1 µl of primer mix, 2 µl of cDNA and 2 µl of nuclease free water to complete the reaction volume. Conditions of the Multiplex-PCR were as follows: 95°C for 15 min, followed by 7 cycles of 95°C for 30 sec, 56°C for 45 sec, decreasing −.5°C per cycle and 72°C for 30 sec, and another 38 cycles of 95°C for 30 sec, 53°C for 1 min and 30 sec, and 72°C for 30 sec, with a final extension of 60°C for 5 min. PCR products were run on an electrophoresis gel and, for samples amplifying a product with the expected size, PCR products were amplified again to incorporate Illumina indexes using the KAPA HiFi HotStart ReadyMix (Kapa Biosystems, Cape Town, South Africa) recommended for amplicon library preparation by Illumina. Each PCR product included its own, unique, index sequence. PCR reaction volume was 14 µl and included: 7 µl 2x KAPA HiFi HotStart ReadyMix, 1.4 µl of mixed indexing primer at 10 mM, 2.8 µl of PCR product and 2.8 µl of nuclease free water. Indexing thermal cycling conditions were 95°C for 3 min, followed by 8 cycles of 95°C for 30 sec, 55°C for 30 sec, 72°C for 30 sec, with a final extension of 72°C for 5 min. Index PCR products were visualized by electrophoresis in agarose gel. Positive samples were purified using 0,8X AMPure®XP beads, quantified in Epoch spectrophotometer (Agilent, Santa Clara, CA, USA) and normalized at 20 nM before pooled. Final library was validated in the TapeStation System (Agilent, Santa Clara, CA, USA) using High Sensitivity D1000 Screen Tape assay and purified again using 0,68X AMPure®XP beads to clean up fragments around 250 bp. After this clean up, the pool was quantified in Qubit (ThermoFisher, Waltham, MA, USA) and in the pool concentration was tested by a qPCR using KAPA Library Quantification Kit for Illumina platforms (Kapa Biosystems, Cape Town, South Africa). Finally, double indexed PCR amplicons were sequenced in an Illumina MiSeq System using MiSeq V2 500-cycle reagent kit with paired-end reads (Illumina, San Diego, CA, USA). Sequences obtained by NGS were aligned by read pairs, purified, and cleaned using ObiTools software [46] to align and remove any sequence errors, primer sequences and fragments with less than 380 bp and 100 reads. After the first bioinformatic analysis with ObiTools, these sequences were analysed by performing a phylogenetic tree with the same software described above. In this case, the analysed fragment was 381 bp and the phylogenetic tree was generated using the maximum likelihood method with General Time Reversible model [47], which had the lowest values of cAIC and BIC. Bootstrap confidence limits were calculated based on 1000 replicates, branch numbers in the tree indicate Bootstrap results (Figure 3).

#### 2.4.3. SARS-CoV-2 specific real-time RT-PCR

All positive samples in the PCRs described above were analysed using a specific real-time RT-PCR for SARS-CoV-2 developed by the Center for Disease Control and Prevention (CDC). Primers and probes sequences are described in Table 2, which were used to individually amplify two targets of virus nucleocapsid gene (N1 and N2). All reactions were carried out on the CFX96 Touch Real-Time PCR Detection System using the SuperScript™ III One-Step RT-PCR System with Platinum™ Taq DNA Polymerase reagent kit (ThermoFisher, Waltham, MA, USA). Volume reaction was adjusted at 15 µl, and included 7.5 µl of 2X Reaction Mix, 0.32 µl of Magnesium Sulphate (5 mM), 0.8 µl of SuperScriptTM III RT PlatinumTM Taq Mix, 1 µl of mix of primers and probes (10 µM), 2 µl of RNA and 3.38 µl of nuclease free water. Conditions of real-time RT-PCR were as follows: reverse transcription was carried out at 55°C for 20 min, followed by polymerase activation and first denaturation at 95°C for 3 min and by 50 cycles split in two steps: 95°C for 3 sec, 55°C for 30 sec.

## 3. Results

At least one of the α-CoV variant was detected by generic coronavirus real-time RT-PCR or conventional multiplex PCR in all sampled locations except in one site (location C), while a β-CoV was detected only in one place (location N) (Figure 1). Nineteen bat species out of 22 sampled were positive for the presence of coronavirus, being *Barbastella barbastellus, Plecotus austriacus* and *Rhinolophus mehelyii* the only bat species that showed no positive results (Figure 1).

The generic real-time RT-PCR revealed 102 samples positives for coronavirus out of the 194, detected in 14 locations and in 18 bat species. Four out of the 102 positive samples were sequenced for the fragment of 118 bp of ORF1b region, and all were isolated from the species *M. schreibersii*, two from location J, one from location R and one from location N (Figure 1). These sequences were compared with the sequences available in the GenBank database and showed a homology between 86% and 96%. In the phylogenetic tree, these sequences clustered in two groups, samples with ID: 40, 42, and 82 clustered with α-CoV, and only the sequence from sample ID 68 was clustered with sarbecoviruses, within the β-CoV (Figure 2). The accession numbers of these new four sequences are ON153217-ON153220.

Conventional PCR revealed 15 samples positive for coronavirus out of 194, detected in seven locations and in five bat species. Five samples were discarded as the coverage of sequences obtained was less than 100 reads. For the remaining 10 samples, 18 distinct viral sequences were obtained, and ranged between 1 and 5 sequences per sample (Table 3). One same viral sequence was obtained from eight distinct samples (ID 42, 78, 79, 82, 85, 87, 97 and 123; Figure 3), while five different sequences were obtained in the same sample, ID 83 and 122 (Figure 3). The accession numbers of these sequences are ON101709-ON101723. Sequences from positive samples ID 78, 79 and 85 are homologous to each other (GenBank accession number ON101717). Sequences detected in samples ID 82 and 83 (Seq_1) are also homologous to each other (Figure 3 & Table 3; GenBank accession number ON101719). All studied sequences were from four locations of the same province, Murcia, belonging to four bat species: *Hypsugo savii, M. schreibersii, Myotis capaccini* and *Pipistrellus kuhlii*, and showed homology between 86% and 100% to the sequences from the GenBank database (Figure 1). In the phylogenetic tree, the 18 sequences clustered in the α-CoV group (Figure 3).

**Table 3.** NGS sequencing results.

Reads	Sample ID																	Length (bp)	GenBank accession number
	ID 1	ID 2	ID 42	ID 58	ID 78	ID 79	ID 82	ID 83	ID 85	ID 87	ID 97	ID 122	ID 123	ID 180	ID 182	ID APC	ID HPC		
2532	0	1	0	0	376	228	0	1	1827	97	0	0	0	2	0	0	0	382	ON101717
2351	0	0	0	0	0	0	0	0	0	0	0	0	0	0	0	2351	0	382	-
2220	0	0	0	0	0	0	0	0	0	0	0	0	0	0	0	0	2220	382	-
1992	0	0	0	0	0	0	0	0	0	0	1992	0	0	0	0	0	0	382	ON101716
1274	0	0	0	0	0	0	0	1274	0	0	0	0	0	0	0	0	0	381	ON101723
1270	1	0	0	0	0	0	0	1269	0	0	0	0	0	0	0	0	0	382	ON101721
1168	0	0	1167	0	0	0	0	0	0	0	0	1	0	0	0	0	0	382	ON101714
941	0	0	0	0	0	0	808	133	0	0	0	0	0	0	0	0	0	382	ON101719
599	0	0	0	0	0	0	0	0	0	598	0	0	0	0	1	0	0	382	ON101718
347	0	0	0	0	0	0	0	0	0	0	0	347	0	0	0	0	0	381	ON101711
308	0	0	0	0	0	0	0	0	0	0	0	308	0	0	0	0	0	381	ON101709
266	0	0	24	0	0	0	0	229	13	0	0	0	0	0	0	0	0	382	ON101720
215	0	0	0	0	0	0	0	0	0	0	0	215	0	0	0	0	0	382	ON101710
193	0	0	0	0	0	0	0	0	0	0	0	193	0	0	0	0	0	381	ON101713
175	0	0	11	0	0	0	0	156	8	0	0	0	0	0	0	0	0	381	ON101722
147	0	0	0	1	0	0	0	0	0	0	1	0	144	1	0	0	0	382	ON101715
134	0	0	0	0	0	0	0	0	0	0	0	134	0	0	0	0	0	382	ON101712
45	0	0	0	0	0	0	0	0	45	0	0	0	0	0	0	0	0	382	-
19	0	0	19	0	0	0	0	0	0	0	0	0	0	0	0	0	0	382	-
17	0	0	0	0	0	0	0	0	0	0	0	9	8	0	0	0	0	381	-
11	0	0	0	0	0	0	0	0	0	0	0	10	1	0	0	0	0	381	-
11	0	0	11	0	0	0	0	0	0	0	0	0	0	0	0	0	0	381	-

The SARS-CoV-2 PCR showed 12 positives out of 194 samples. These PCR products were less than 100 bp and the information provided by sequencing of these fragments was limited. Nevertheless, all positive samples for this PCR were also positive in the real-time RT-PCR described above.

## 4. Discussion

Bats play a key role in both natural and anthropized ecosystems with fundamental contributions to human wellbeing, such as feeding on agricultural pests, pollination, and ecological seed dispersal [48,49]. They also play an important role in the health of these ecosystems and are intrinsically intertwined with animal and human health as approached in the One Health concept [50]. Bats are considered the ancestral hosts of many pathogenic viruses from different families, including lyssaviruses, henipaviruses, filoviruses, bunyaviruses and coronaviruses [51]. Recently, it has been shown that novel coronavirus closely related to SARS-CoV-2 may be transmitted by bats to other species and then passed on to humans [11–14].

Among the 31 known native bat species in the Iberian Peninsula, a total of 22 were sampled from the West to the East of mainland Spain. Of the 194 bats analysed, 53% tested positive for coronaviruses in at least one of the three PCR tests performed. These results suggested a high prevalence of these viruses in bats from the Iberian Peninsula. Viral detection by conventional PCR was lower than that obtained by the generic real-time PCR, possibly due among other reasons, to the different sensitivity of the techniques and size of the amplified fragments. The real-time RT-PCR analysis showed several peaks in the melting curve with differences of 0.5°C between positive samples and the positive control (data not shown), suggesting that different variants of coronavirus were amplified. Indeed, sequencing and phylogenetic analysis revealed that these viruses were classified as α-CoV and β-CoV and, according to the current ICTV classification system for coronaviruses [52], as Minunacovirus, Myotacovirus, Pedacovirus and Sarbecovirus (Figures 2 and 3).

The success of the conventional Sanger sequencing (only 4/102 samples were successfully sequenced) might be affected by the small fragment size, together with the likely amplification of more than one strain from a single sample, suggesting that multiple coronavirus strains are present in the same sample. Additionally, this was further confirmed by the conventional multiplex PCR, where more than one α-CoV variant was obtained from same sample (bats ID 83 and 122) with up to 5 sequence variants detected. Recombination is one of the major drivers of virus evolution; considering the rapid evolution and complexity of these viruses with sufficient capacity to generate new genomes, the coexistence of different coronaviruses in the same bat enables the existence of recombination between them inside the animal to potentially become a risk for other animals and humans [53]. Furthermore, these results confirmed the ability of bats to resist and tolerate viruses with low viraemia and the absence of clinical symptoms [51], which has been observed even in experimentally infected bats with highly lethal viruses such as henipavirus [54,55], MERS-CoV [56], Ebola virus [57], and Marburg virus [58]. However, future cohort and long-term studies must determine the impact of coronaviruses, in terms of infection and evolution of disease, in native bats from the Iberian Peninsula.

Bats live in colonies of a few to thousands of individuals and possess a high longevity compared to other mammals of their size [59]. This, together with their ability to fly, explains why bats are exposed to a wide variety of viruses, which influences the evolution of their immune system making them resistant to different viruses [60]. The first line of defence against any virus is the innate immune response, which is also related to bat flying ability, which requires metabolic adaptation to rapid increases in activity, body temperature and associated molecular damage. Therefore, the ability to fly could be related to bat capacity to regulate the associated inflammatory processes, which would also give them an adaptive advantage in resistance to pathogens [61].

**Figure 4.**
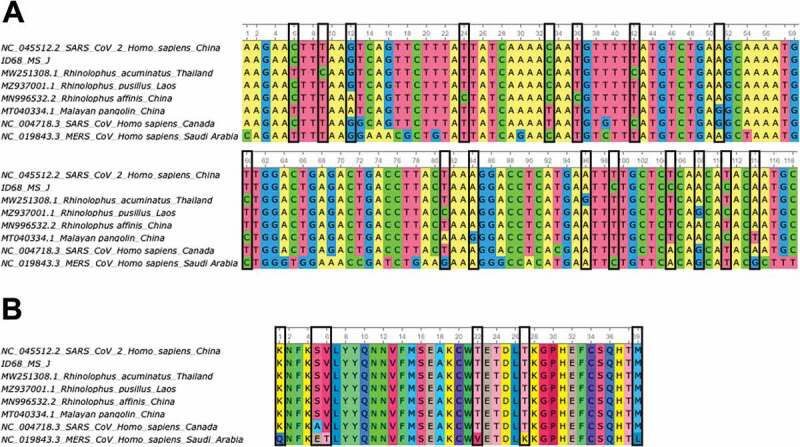
A) Alignment of partial ORF1b gene sequences (118 bp) of some bat-related SARS-CoV-2 (MN996532.2, MZ937001.1, MW251308.1) and pangolin (MT040334.1) coronavirus isolates, including the SARS-CoV-2 Wuhan-Hu-1 (NC_045512.2), SARS-CoV (NC_004718.3) and MERS-CoV (NC_019843.3) reference sequences and the sequence obtained in this study ID 68 (ON153220). Nucleotide changes between bat and pangolin isolates related to SARS-CoV-2 with the reference sequence for this virus are highlighted in black boxes. B) Amino acid alignment (39 aa) of partial ORF1b gene sequences (118bp) described in section A of this figure. Amino acid changes between bat and pangolin isolates related to SARS-CoV-2 with the reference sequence for SARS-CoV and MERS-CoV are highlighted in black boxes.

Most of the coronaviruses detected in this study belong to α-CoV genus but one of them was classified as β-CoV through phylogenetic analysis. In this case, the sample ID 68 was positive for two of the molecular tests, the real-time RT-PCR [44] and the specific SARS-CoV-2 PCR (https://www.cdc.gov/coronavirus/2019-ncov/lab/virus-requests.html). Although it was positive for SARS-CoV-2 PCR, it cannot be confirmed since the information provided by this PCR is very limited because it amplifies a short fragment of SARS-CoV-2 genome and thus confirmation would require deepest sequencing of the genome. However, the sequence obtained from the real-time RT-PCR for this sample supported that this is a coronavirus of the β-CoV genus, closely related to SARS-CoV-2 (Figures 2 and 4). Subsequently, the rapid evolution and complexity of these viruses with sufficient capacity to generate new genomes through co-infection and recombination may facilitate cross-species transmission [51,62]. Furthermore, our results provide additional support for the potential risk of SARS-CoV re-emergence from viruses currently circulating in bat populations [63].

Bats are reservoirs for a wide range of different viruses including coronaviruses where they are putative precursors of pandemic-associated viruses SARS-CoV [4–6], MERS-CoV [7–10] and more recently SARS-CoV-2 [11–14]. The risk of emergence of zoonotic pathogens in humans depends on the interactions between humans and infected animals, reservoirs and/or vectors, or their environment. Knowledge of the causal relationships between the human-animal interface and the emergence of pathogens in humans is still incomplete. However, the climate change, land use change (deforestation, urbanization, crop intensification), habitat alterations, changes in animal production management and in food and water availability, and other human activities favour and increase in the interactions between humans and other animals [64]. As with other viruses prior to the SARS-CoV-2 pandemic, the emergence of numerous viruses has been attributed to increased interspecific contact between humans and wildlife following increased bushmeat hunting and invasion on undisturbed habitats [64,65].

In conclusion, the results of this study encourage research to better understand and characterize coronavirus genetic variability and natural hosts. Extensive viral sampling in multiple bat species and other wild and domestic animals in many different locations, epidemiological and ecological contexts is required to improve control and prevention measures against newly associated emerging zoonotic diseases.
